# Supplementary material for: A study of the extraordinarily strong and tough silk produced by bagworms
Source: Nat Commun. 2019 Apr 1;10:1469. doi: 10.1038/s41467-019-09350-3 (PMC6443776; doi:10.1038/s41467-019-09350-3)
Supplement: Supplementary file 1 — Supplementary Information [file 41467_2019_9350_MOESM1_ESM.pdf]

<u>Supplementary information PDF</u>

A Study of the Extraordinarily Strong and Tough Silk Produced by Bagworms

Yoshioka et al.

Amino acids	mol / %
Glycine	42.3
Alanine	39.4
Valine	2.0
Leucine	0.7
Isoleucine	0.4
Serine	6.7
Threonine	0.9
Aspartic acid	1.4
Glutamic acid	1.8
Arginine	0.4
Histidine	0.2
Lysine	0.9
Tyrosine	1.8
Phenylalanine	0.4
Proline	0.8
Cystein	N.D.
Methionine	N.D.
Total	100.1

Supplementary Fig. 1 Amino acid composition of *E. variegate* bagworm silk.

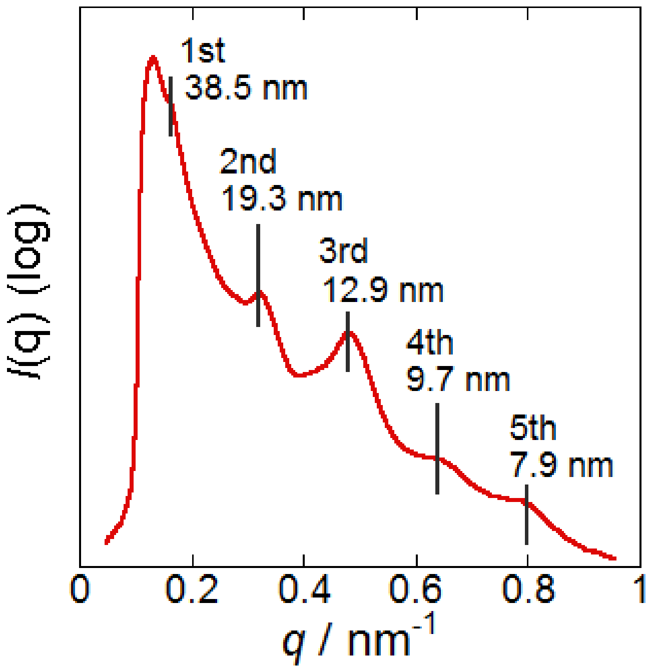

Supplementary Fig. 2 The meridional SAXS *q* vs. *I*(*q*) profile of *E. variegata* bagworm silk, corresponding to the *q* vs. $I(q)q^2$ profile shown in Fig. 3d.

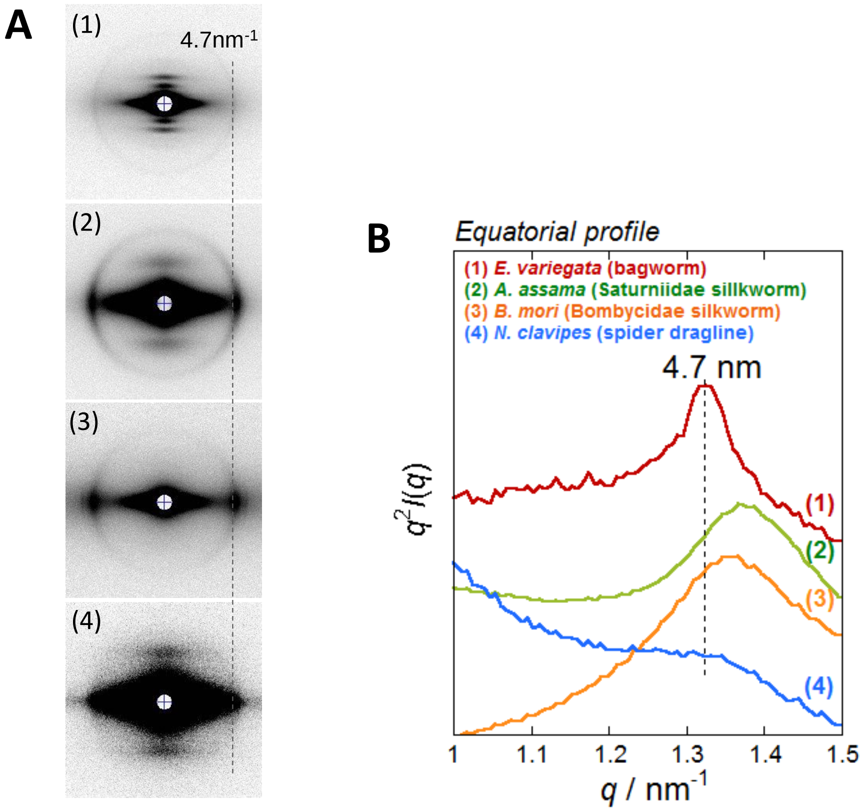

C

	E. variegata (Bagworm)	A. assama (Saturniidae)	B. mori (Bombycidae)	N. Clavipes (Spider dragline)
Thickness / nm	155	35	41	37

Supplementary Fig. 3 **A**, 2d-SAXS patterns of (1) *E. variegata* bagworm silk, (2) *A. assama* saturniidae silkworm silk[1], (3) *B. mori* bombycidae silkworm silk, and (4) *N. clavipes* spider dragline silk, and **B**, the equatorial q vs. $I(q)q^2$ profiles for each SAXS pattern. **C**, Table of nanofibril bundle thickness estimated by Scherrer equation.

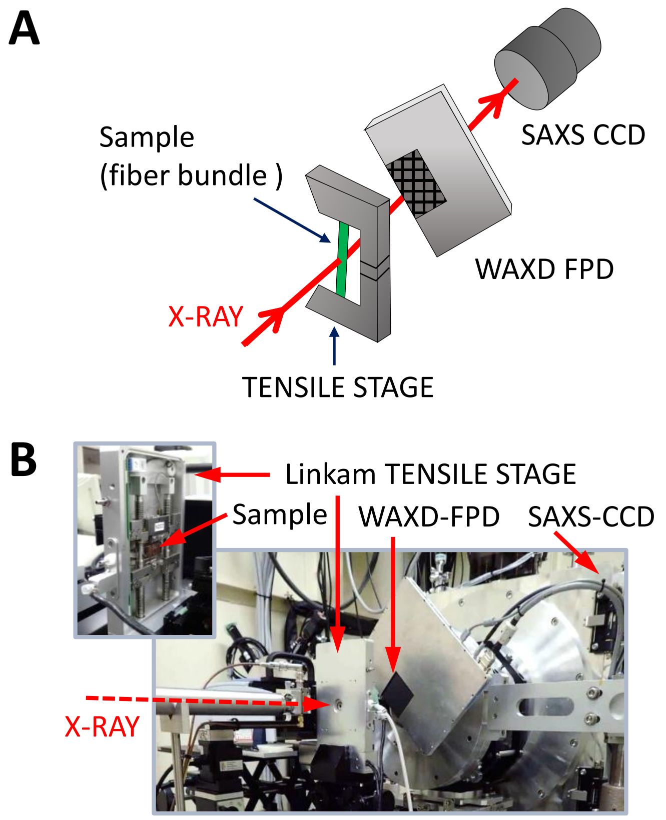

Supplementary Fig. 4 **A,** Schematic drawing and **B,** actual view of the experimental setup for the time-resolved simultaneous measurement of tensile test and synchrotron SWAXS, performed at SPring-8 beam line 40B2.

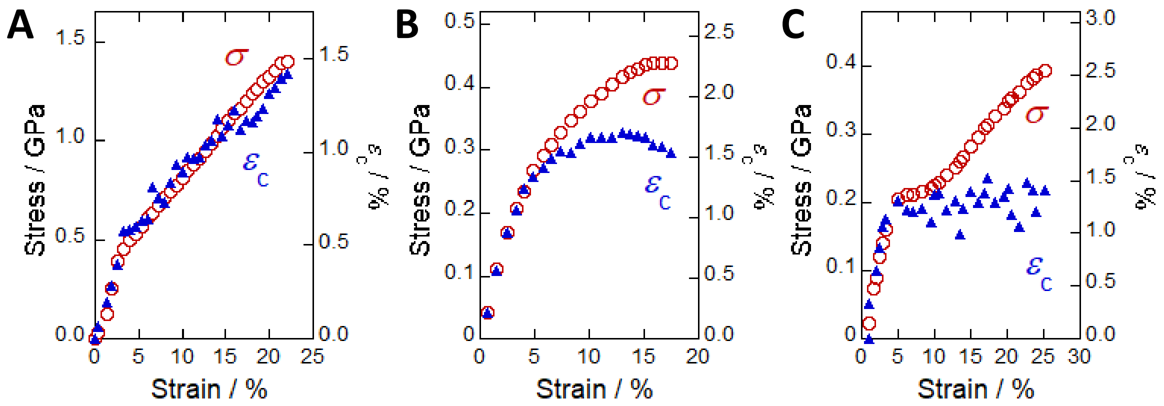

Supplementary Fig. 5 The changes of tensile stress and crystal strain (ε_c) against the bulk strain (ε) in the time-resolved simultaneous synchrotron X-ray analysis for **A**, the *E. variegata* bagworm silk, **B**, *B. mori* bombycidae silkworm silk and **C**, *A. assama* saturniidae silk, respectively. The changes of bulk strain and crystal strain against the tensile stress for them are shown in Fig. 6.

A

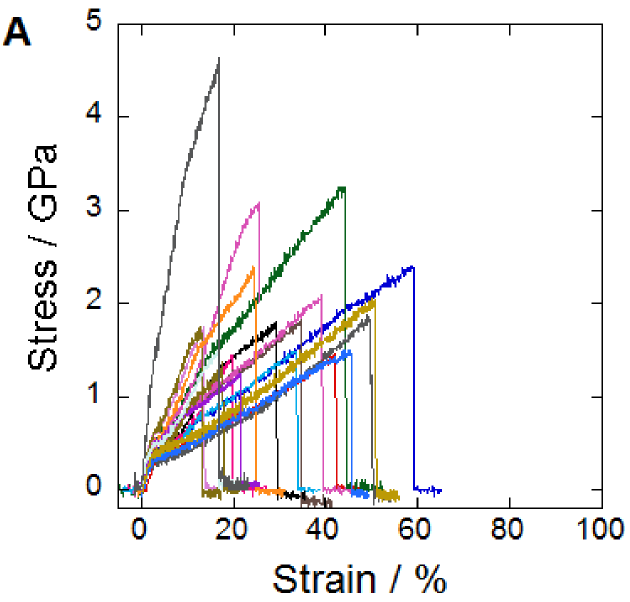

B

	No.	Young's modulus (GPa)	Fracture strength (GPa)	Extensibility (%)	Toughness (MJ/m^3)
	1	34.40	1.85	49.7	442.74
	2	27.09	2.02	39.3	466.56
	3	16.15	1.82	34.7	355.71
	4	31.93	3.02	27.5	385.43
	5	20.43	1.43	20.0	141.65
	6	20.76	1.77	29.3	292.26
	7	28.29	3.20	44.0	797.34
	8	20.96	2.33	59.2	780.65
	9	14.25	1.43	41.8	331.40
	10	28.09	1.46	34.0	428.12
	11	58.48	4.60	17.0	472.51
	12	27.37	1.47	17.0	139.10
	13	20.83	1.22	21.0	172.77
	14	24.29	2.01	51.0	535.44
	15	29.74	1.49	46.0	373.45
	16	27.39	2.30	25.0	327.27
	17	38.09	1.76	13.0	126.07
	18	33.19	1.37	18.0	130.40
	19	32.60	1.67	14.0	217.40
Average		28.12	2.01	31.66	364.01
Standard deviation (SD)		9.20	0.80	13.74	192.05
Standard error of mean (SEM)		2.11	0.18	3.15	44.06

Supplementary Fig. 6 All the SS-curves **A,** and mechanical properties **B,** of bagworm silks measured in this study (averaged in Table 3).

Supplementary Note 1

A simple calculation of crystalline phase length (23 nm) / long period (39 nm) × 100 (%) gives an apparent crystallinity of ~59%. However, this is only true under the assumption that the bagworm silk protein is composed only of H-Fib proteins and the H-Fib protein is made up of the determined tandem sequence motifs listed in Fig. 1. However, the motifs we determined are probably not all, and it was also clarified that the bagworm silk contains not only H-Fib, but also at least one kind of light-chain fibroin (we will be reporting this in the near future). The crystallinity evaluated by WAXD analysis (~45%) is considered to be more reliable.

Supplementary Note 2

The crystal modulus is defined as the slope of the stress‑crystal strain plot, where the crystal strain (or the degree of crystal deformation) can be measured by WAXD analysis. When the sample is a semi-crystalline polymeric fibre, the crystal modulus is evaluated under the assumption that the applied stress distributes to the crystal and amorphous parts homogeneously. Thus, under this assumption the resultant crystal modulus should show a true modulus of single crystal. However, in actual this homogeneous stress distribution is not considered to occur, and therefore, the crystal modulus evaluated is called the ‘apparent crystal modulus’. The accuracy of this apparent crystal modulus is strongly affected by the degree of heterogeneous stress distribution[2].

Supplementary Note 3

In this study, we showed that the apparent crystal modulus of β-sheet silk crystals differs drastically depending on the species. There are mainly two possible reasons for this. One is that the value of the true crystal modulus in each silk differs drastically. The other is that the value of the true crystal modulus is approximately similar for each silk, but the degree of heterogeneous stress distribution differs drastically. Judging from the similar WAXD results obtained from each silk, the latter possibility is believed to be more significant.

Supplementary References

1. Yoshioka, T., Kameda, T. X-ray scattering analyses quantitatively revealed periodic hierarchical structure of polyalanine β-sheet and non-polyalanine amorphous domains in Antheraea assamensis (Muga) silk. *J. Silk Sci. Tech. Jpn.* **27**, 95-101 (2019).
2. Nakamoto, S., Tashiro, K., Matsumoto, A. Quantitative evaluation of stress distribution in bulk polymer samples through the comparison of mechanical behaviours between giant single-crystal and semicrystalline samples of poly(trans-1,4-diethyl muconate). *J. of Polym. Sci., Part B: Polym. Phys.,* **41**, 444-453 (2003).
